# Identification of fetal unmodified and 5-hydroxymethylated CG sites in maternal cell-free DNA for non-invasive prenatal testing

**DOI:** 10.1186/s13148-020-00938-x

**Published:** 2020-10-20

**Authors:** Juozas Gordevičius, Milda Narmontė, Povilas Gibas, Kotryna Kvederavičiūtė, Vita Tomkutė, Priit Paluoja, Kaarel Krjutškov, Andres Salumets, Edita Kriukienė

**Affiliations:** 1 Department of Biological DNA Modification, Institute of Biotechnology, Vilnius University, Saulėtekio av. 7, 10257 Vilnius, Lithuania; 2 Competence Centre On Health Technologies, Teaduspargi 13, 50411 Tartu, Estonia; 3 Department of Obstetrics and Gynaecology, Institute of Clinical Medicine, University of Tartu, L. Puusepa 8, 50406 Tartu, Estonia; 4 Department of Obstetrics and Gynecology, University of Helsinki and Helsinki University Hospital, HUS, PO Box 140, 00029 Helsinki, Finland; 5 Estonian Genome Center, Institute of Genomics, University of Tartu, Riia 23b, 51010 Tartu, Estonia; 6 Faculty of Medicine, University of Helsinki, 00014 Helsinki, Finland; 7 Institute of Biotechnology, Vilnius University, Saulėtekio av. 7, 10257 Vilnius, Lithuania

**Keywords:** NIPT, Fetal trisomy, Down syndrome, Cell-free DNA, DNA modification, 5-Hydroxymethylcytosine, Covalent labeling

## Abstract

**Background:**

Massively parallel sequencing of maternal cell-free DNA (cfDNA) is widely used to test fetal genetic abnormalities in non-invasive prenatal testing (NIPT). However, sequencing-based approaches are still of high cost. Building upon previous knowledge that placenta, the main source of fetal circulating DNA, is hypomethylated in comparison to maternal tissue counterparts of cfDNA, we propose that targeting either unmodified or 5-hydroxymethylated CG sites specifically enriches fetal genetic material and reduces numbers of required analytical sequencing reads thereby decreasing cost of a test.

**Methods:**

We employed uTOPseq and hmTOP-seq approaches which combine covalent derivatization of unmodified or hydroxymethylated CG sites, respectively, with next generation sequencing, or quantitative real-time PCR.

**Results:**

We detected increased 5-hydroxymethylcytosine (5hmC) levels in fetal chorionic villi (CV) tissue samples as compared with peripheral blood. Using our previously developed uTOP-seq and hmTOP-seq approaches we obtained whole-genome uCG and 5hmCG maps of 10 CV tissue and 38 cfDNA samples in total. Our results indicated that, in contrast to conventional whole genome sequencing, such epigenomic analysis highly specifically enriches fetal DNA fragments from maternal cfDNA. While both our approaches yielded 100% accuracy in detecting Down syndrome in fetuses, hmTOP-seq maintained such accuracy at ultra-low sequencing depths using only one million reads. We identified 2164 and 1589 placenta-specific differentially modified and 5-hydroxymethylated regions, respectively, in chromosome 21, as well as 3490 and 2002 Down syndrome-specific differentially modified and 5-hydroxymethylated regions, respectively, that can be used as biomarkers for identification of Down syndrome or other epigenetic diseases of a fetus.

**Conclusions:**

uTOP-seq and hmTOP-seq approaches provide a cost-efficient and sensitive epigenetic analysis of fetal abnormalities in maternal cfDNA. The results demonstrated that T21 fetuses contain a perturbed epigenome and also indicated that fetal cfDNA might originate from fetal tissues other than placental chorionic villi. Robust covalent derivatization followed by targeted analysis of fetal DNA by sequencing or qPCR presents an attractive strategy that could help achieve superior sensitivity and specificity in prenatal diagnostics.

## Background

Down syndrome, the trisomy of chromosome 21 (T21), is the most common incurable chromosomal aneuploidy in live born infants and is associated with physical and mental disability [1]. Invasive diagnostic procedures such as amniocentesis, chorionic villus sampling or cordocentesis are currently used to confirm the diagnosis of T21, commonly by a fetal karyotyping. Although the safety of invasive procedures has been improving, a risk of fetal loss (0.5 to 1% for chorionic villus sampling and amniocentesis) and follow-up infections still remain [2]. Hence, to reduce the number of invasive diagnostic procedures, non-invasive and highly confident prenatal screening tests are still required.

Since the discovery of fetal genomic material in the form of circulating cell-free fetal DNA (cffDNA) in the blood plasma of pregnant women [3], many efforts have been made to employ cffDNA for non-invasive prenatal testing (NIPT) of fetal chromosomal aneuploidies. However, the detection of cffDNA in maternal blood circulation has represented a considerable challenge. Since cffDNA comprises only a 6–10% fraction of the total maternal cfDNA in first and second trimester pregnancies [4, 5], this can often interfere with the analysis of fetal nucleic acids. The issue of the low abundance of cffDNA can be overcome by evaluating the dosage of chromosome 21 from the ratios of polymorphic alleles in the placenta-derived nucleic acid molecules [6]. However, this approach can only be applied to a subset of the population, when fetuses are heterozygous for the targeted polymorphisms.

Massive parallel sequencing (MPS) has been employed for the detection of fetal aneuploidy through measuring the dosage of any chromosome in maternal plasma. Previous reports have indicated that cffDNA is shorter than its maternal counterpart [7–9]. Therefore, MPS has been combined with size fractionation prior to sequencing or in silico filtering of cfDNA to enrich for fetal DNA fragments. However, even though MPS has been widely used in commercial prenatal testing, such an approach which requires deep sequencing, increases the cost of medical service.

An alternative approach to improve the cost-effectiveness of NIPT is to preferentially target fetal DNA sequences based on their DNA modification differences between maternal cfDNA and cffDNA. Bisulfite conversion, methyl-DNA immunoprecipitation (MeDIP) and methylation sensitive restriction digestion have already been employed for the identification of fetal-specific differentially methylated regions that can be used for the detection of fetal aneuploidies [10–12]. Although bisulfite conversion enables analysis of the methylation status of each CG site [13–17], it reinforces degradation of cfDNA, further reducing the amount of cffDNA available for fetal-specific methylome analysis. Furthermore, screening genomes by whole-genome bisulfite sequencing is technologically demanding and extremely expensive leading to an unnecessary increase in cost of NIPT. The methyl-DNA immunoprecipitation and methylation sensitive restriction digestion enrich hypermethylated fetal-specific DNR regions [10, 11, 18, 19]. However, methylation sensitive restriction digestion is inherently limited by the sequence-specificity of available enzymes which restricts the number of regions suitable for testing. MeDIP enrichment is biased to highly methylated sequences [20] and thus, the potential diagnostic informativeness of the low CG density regions or less methylated sequences might be lost.

Examination of the differential methylation between placenta and maternal blood uncovered significant placental hypomethylation relative to cfDNA of non-pregnant women [13]. These hypomethylated regions have low CG and gene density and thus could be poorly covered by affinity enrichment methods, such as MeDIP. Moreover, since the unmodified CG fraction represents a smaller portion of the human genome (20–30% of CGs are unmethylated in human tissues [21]), its targeted analysis could be more relevant for the cost-effective NIPT. Therefore, further technological advances are necessary for the identification of effective and stable fetal-specific biomarkers for aneuploidy diagnostics.

In recent years, covalent derivatization has been adapted for epigenome-wide studies of various cytosine modifications [22–25]. Here, we applied for the first time the covalent derivatization of unmodified CG sites (uCGs) in maternal cfDNA for identification of fetal-derived genomic regions. In addition, we detected 5-hydroxymethylcytosine (5hmC) in placental CV DNA samples, and for the first time demonstrated its utility for detection of fetal karyotype. We firmly believe that both approaches of covalent targeting combined with sequencing and innovative bioinformatic data analysis or qPCR can vastly reduce the cost and turnaround time, increasing the availability of NIPT.

## Results

### Genome-wide uCG and 5hmCG patterns suggest strong fetal contribution to maternal cfDNA

We sought to identify DNA fragments of fetal origin in maternal cfDNA by analyzing unmodified and 5-hydroxymethylated cytosines located in CG dinucleotides (uCG and 5hmCG, respectively). In order to test the feasibility of 5hmC analysis in cfDNA, we analyzed global amounts of 5hmC in two trophoblast-enriched CV tissue and three blood DNA samples by a HPLC–MS/MS assay and found them to be higher in CV samples (0.021 ± 0.002% of total cytosine) than in blood DNA (0.012 ± 0.002% of total cytosine).

For profiling of uCGs and 5hmCGs we employed our recently developed uTOP-seq and hmTOP-seq strategies, respectively, which assess the modification status of genomic CG sites through selective covalent coupling of a priming oligonucleotide to azide-modified CGs and their subsequent sequencing [24, 25]. By leveraging the high robustness of covalent derivatization and the sensitivity of such targeted sequencing we successfully adapted both strategies for the analysis of nanogram quantities of cfDNA.

We constructed uCG and 5hmCG maps of CV tissue samples (CVS; n = 7 of uCG and n = 3 of 5hmCG signals) and cfDNA samples (n = 38 maps in total). cfDNA samples consisted of non-pregnant controls (NPC; uCG n = 7 and 5hmCG n = 7) and pregnant women carrying healthy (uCG n = 8 and 5hmCG n = 7) or T21 fetuses (uCG n = 5 and 5hmCG n = 4). Fetal sex was approximately equally distributed across sample groups (Additional file 1: Table S1).

**Fig. 1:**
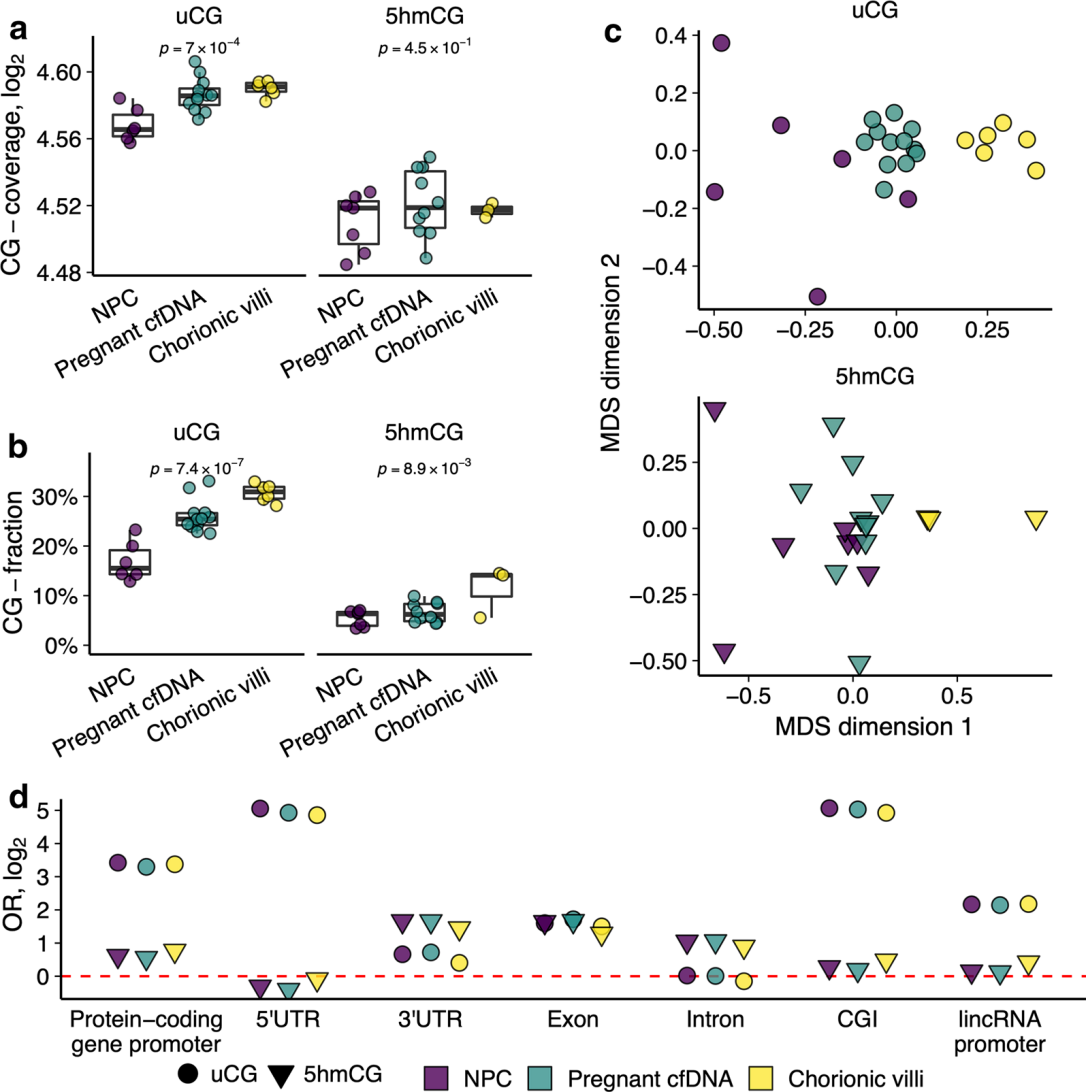
uTOP-seq and hmTOP-seq analysis of CV and cfDNA samples reveals a contribution of CV tissue to maternal cfDNA. **a** Total CG-coverage in cfDNA and CV tissue sample groups for the uCG and 5hmCG signals. Total log2 transformed sequencing coverage of autosomes was computed for each sample and ANOVA was used to test for differences in distributions across sample groups. **b** CG-fraction in cfDNA and CV tissue sample groups for the uCG and 5hmCG data. Fraction of CGs covered by at least one read to the total number of CGs was computed for each sample and ANOVA was used to test for differences in distributions across sample groups. **c** Non-metric multidimensional scaling plots of genome-wide signal distribution using two dimensions for uCG and 5hmCG data (using samples after the outlier removal). **d** Odds ratio (OR) for enrichment of uCG and 5hmCG data across genomic elements (*p* < 1.6 × 10^–10^). The genome was divided into 1,000-bp windows and total coverage per sample was averaged across sample groups for each window. Fisher’s exact test was used to test whether the windows with the 10% of the strongest signal are enriched with particular genomic elements. CGI, CG island; lincRNA, long intergenic non-coding RNA

In order to test whether uCG and 5hmCG modification differences could distinguish between the sample groups, we first looked at the total sequencing coverage of the uCG and 5hmCG sites (CG-coverage). Mean total uCG coverage was different across the three groups of samples (*p* = 7 × 10^–4^, ANOVA); it was the lowest among NPCs and the highest among CVS. Importantly, the mean total coverage of the pregnant women cfDNA was in between the NPCs and CVS (Fig. 1a). Furthermore, the fraction of identified uCGs covered by at least 1 read showed a similar but even stronger difference among all groups (*p* = 7.4 × 10^–7^, ANOVA; Fig. 1b). For the 5hmCG samples, total coverage was not informative, but the fraction of identified CGs (CG-fraction) increased from NPCs towards CVS (ANOVA *p* = 0.45 and *p* = 8.9 × 10^–3^, respectively; Fig. 1a, b). Non-metric multidimensional scaling transformation of the uCG and 5hmCG profiles confirmed the above observations and revealed substantial modification differences between the sample groups. In both cases, cfDNA samples of pregnant women tended to concentrate between those of NPCs and CVS, suggesting that they might represent a mixture of the latter two groups (Fig. 1c). Considering both higher total coverage and higher fraction of covered CG sites, CV tissue is relatively hypomethylated and also shows increased hydroxymethylation in comparison to cfDNA of NPCs.

Next, we explored the distribution of the identified uCGs and 5hmCGs across genomic elements in all the sample groups. We computed total coverage in 1,000-bp windows genome-wide and tested the enrichment of top 10% most covered windows with genomic elements. As expected, the hypomethylated regions concentrated in CG islands (CGI), promoters of protein coding genes and 5′UTRs, while the highly modified 5hmCGs were mostly observed in 3′UTRs, exons and introns (Fig. 1d). Therefore, both uTOP-seq and hmTOP-seq approaches may provide distinct but complementary data for detection and analysis of cffDNA fragments in maternal circulation.

### Epigenetic profiling determines fetal fraction in cfDNA

**Fig. 2:**
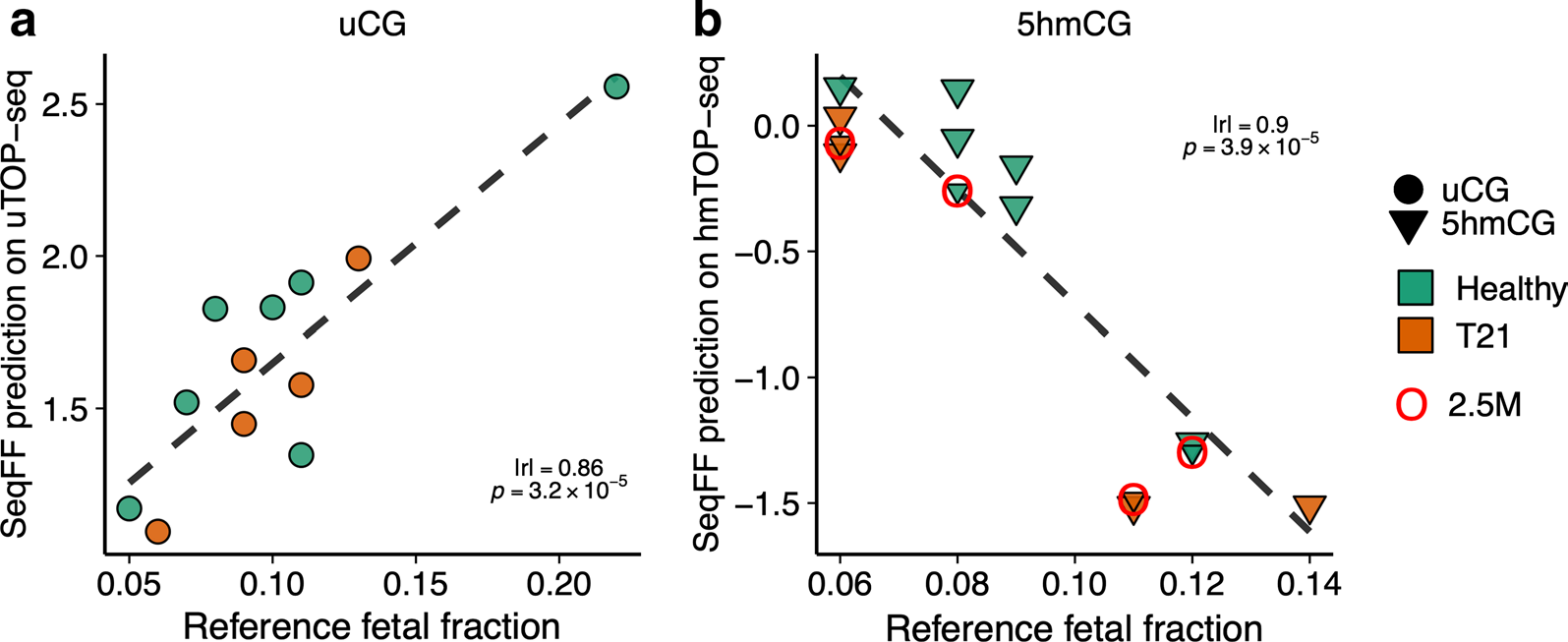
Correlation of the reference fetal fraction and SeqFF prediction from **a** uTOP-seq and **b** hmTOP-seq data indicates the enrichment of cffDNA in maternal cfDNA mixture. Dashed line indicates linear regression. hmTOP-seq samples of shallow sequencing (on average 2.5 million (M) raw reads) are indicated with red circles and were not used in the estimation of Pearson correlation

It is well known that very low or exceptionally high fetal fractions in maternal cfDNA may affect the accuracy of subsequent NIPT tests [26]. Having established that the uTOP-seq and hmTOP-seq signals are higher among pregnant women, we further sought to determine the correlation between the signal strength and fetal fraction. We first used whole genome sequencing and SeqFF algorithm to establish a reference fetal fraction in cfDNA samples. SeqFF is a widely used approach which relies on an increased proportion of short cfDNA fragments, which are more likely of fetal origin, and is independent of fetal sex [27]. We applied SeqFF on the uTOP-seq and hmTOP-seq data and observed high correlation between the predicted and reference fetal fractions (Pearson |r|= 0.86, *p* = 3.2 × 10^–4^ and |r|= 0.9; *p* = 3.9 × 10^–4^, for uCG and 5hmCG, respectively; Fig. 2). Importantly, a simple linear regression revealed that an increase of the reference fetal fraction by 0.01 corresponded to an increase in fetal fraction predicted from uCG profiles by 0.079. For 5hmCG data, the predicted fetal fraction decreased by 0.226 for every 0.01 increase of the reference fraction. Interestingly, an increasing fetal fraction would acquire increasing read counts in uTOP-seq, but decreasing read counts in hmTOP-seq. Such inverse relationship in hmTOP-seq most likely indicates that the regions used by SeqFF are highly enriched in uCGs but depleted in 5hmCGs in cffDNA.

The results indicated that both uTOP-seq and hmTOP-seq enable enrichment of fetal circulating DNA from maternal cfDNA. Importantly, hmTOP-seq may be more sensitive for evaluation of fetal fraction, most likely due to the well-known role of tissue specificity of 5hmC [28]. Consequently, fewer reads would be necessary to provide sensitive analysis of fetal fraction and detection of fetal abnormalities. To further test this hypothesis, we analysed by hmTOP-seq four additional cfDNA samples obtaining on average 2.5 million raw reads for each sample (two healthy and two T21 fetuses). As expected, we observed a very high correlation between reference and predicted fetal fraction in these samples prepared with shallow sequencing depth (Pearson |r|= 0.95, *p* = 0.05; Fig. 2b).

### Chromosome-wide cfDNA uCG and 5hmCG patterns reveal fetal karyotype

We tested whether the total coverage or the fraction of identified CGs (CG-coverage and CG-fraction) in chromosome 21 may be used to detect fetal T21 in pregnant women. As suggested previously [29], for each sample we computed the ratio of total coverage in chromosome 21 and a reference chromosome. We then estimated the mean and standard deviation of these ratios among the healthy pregnancies. We assigned a Z-score to each sample indicating how far away the sample ratio is from the expected ratio of healthy pregnancies. Identical approach was applied for Z-score calculations using CG-fractions. Next, we trained a logistic regression model using Z-score as the independent variable and fetal karyotype as the dependent variable and estimated predictive accuracy of the model using a leave-one-out cross-validation technique. We found that using chromosome 16 for uCG and chromosome 20 for 5hmCG signal as a reference, 100% classification accuracy could be achieved using both CG-coverage and CG-fraction parameters.

**Fig. 3:**
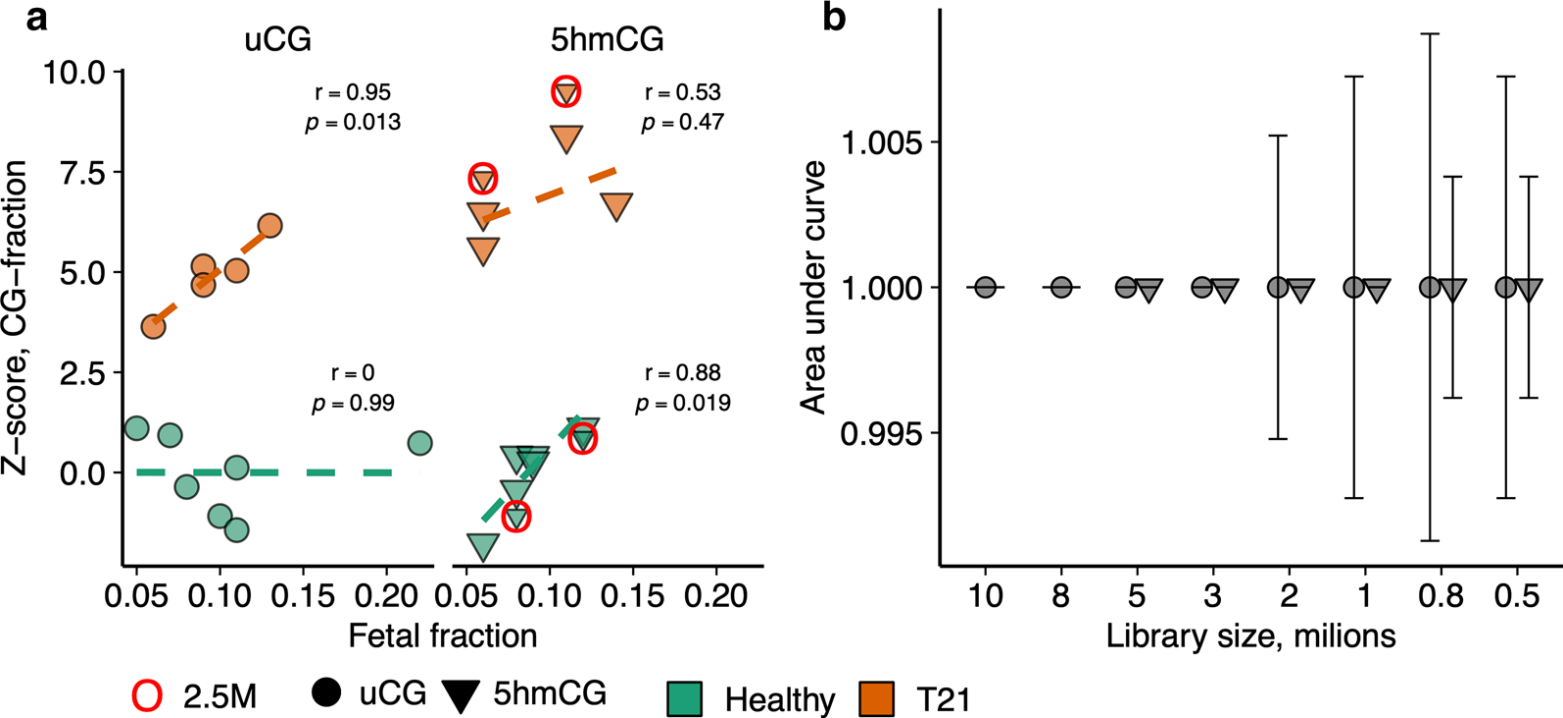
Detection of fetal trisomy T21 using CG-fraction. **a** Effect of fetal fraction on the Z-score indicating fetal karyotype. Normalized CG-fractions in chromosome 21 were used to compute Z-scores. 100% diagnostic accuracy of fetal trisomy T21 independent of fetal fraction was achieved. Dashed lines represent logistic regression fits. hmTOP-seq samples of shallow sequencing (2.5 million raw reads) are indicated with red circles. **b** Effect of reduced library size on classification accuracy. 100% diagnostic accuracy is achieved using 3 million or 1 million of processed sequencing reads of uTOP-seq and hmTOP-seq, respectively. Reads were randomly sampled and karyotype determined using leave-one-out cross-validation. In each cross-validation loop a logistic regression model was built with the Z-scores computed from the normalized CG-fraction within chromosome 21. Error bars indicate the standard deviation from mean AUC across 30 sampling iterations

In uCG data, the Z-score of T21 pregnancies increased with higher fetal fraction consequently widening the gap between healthy and T21 pregnancies (r = 0.95, *p* = 0.013 and r = 0.94, *p* = 0.015 for CG-fraction and CG-coverage, respectively; Pearson correlation), while the Z-scores of the healthy pregnancies did not show a significant trend. In 5hmCG data, there was a significant relationship between Z-score and fetal fraction among healthy but not among T21 pregnancies yet the difference between the two groups was higher than in uCG data (Fig. 3a, Additional file 2: Figure S1a). Notably, irrespective of the computational approach, the fetal fraction did not affect the diagnostic accuracy.

Next, in order to determine the minimal amount of reads necessary for detection of fetal aneuploidy we performed an in silico analysis. We randomly sampled read counts from the original count matrix and repeated the analysis approach described above. For each subset size we performed the sampling 30 times. We found that 5 million processed uTOP-seq reads were necessary to maintain perfect classification (area under curve, AUC = 1) using uCG-coverage in all 30 subsampling iterations (Additional file 2: Figure S1b). Using uCG-fraction the subset could be further reduced to 3 million reads (Fig. 3b). Strikingly, 5hmCG data enabled the reduction of sample size to 1 million reads, maintaining AUC at 1 in all subsampling iterations (Fig. 3b, Additional file 2: Figure S1b).

We validated these calculations experimentally by including the four cfDNA samples of shallow sequencing mentioned above (cfDNA/healthy fetus, n = 2, cfDNA/T21 fetus n = 2; 2.5 million raw reads and 1.3 million processed reads). We combined the low coverage samples with the original ones subsampled down to 1 million reads and observed 100% classification accuracy across all samples (Fig. 3a, Additional file 2: Figure S1a). Thus, this analysis demonstrated a superior diagnostic potential of hmTOP-seq at high and low sequencing depths.

### Placenta-specific differentially modified regions are informative of fetal karyotype

We next sought to identify fetal-specific differentially modified regions (DMRs) that would discriminate between cfDNA of NPCs and both cfDNA of healthy pregnancies and CVS. We partitioned the chromosome 21 into 100 bp-wide non-overlapping windows. For each window we computed the CG-coverage and the CG-fraction and normalized by the CG-coverage and CG-fraction in the reference chromosomes 16 and 20, as above.

**Fig. 4:**
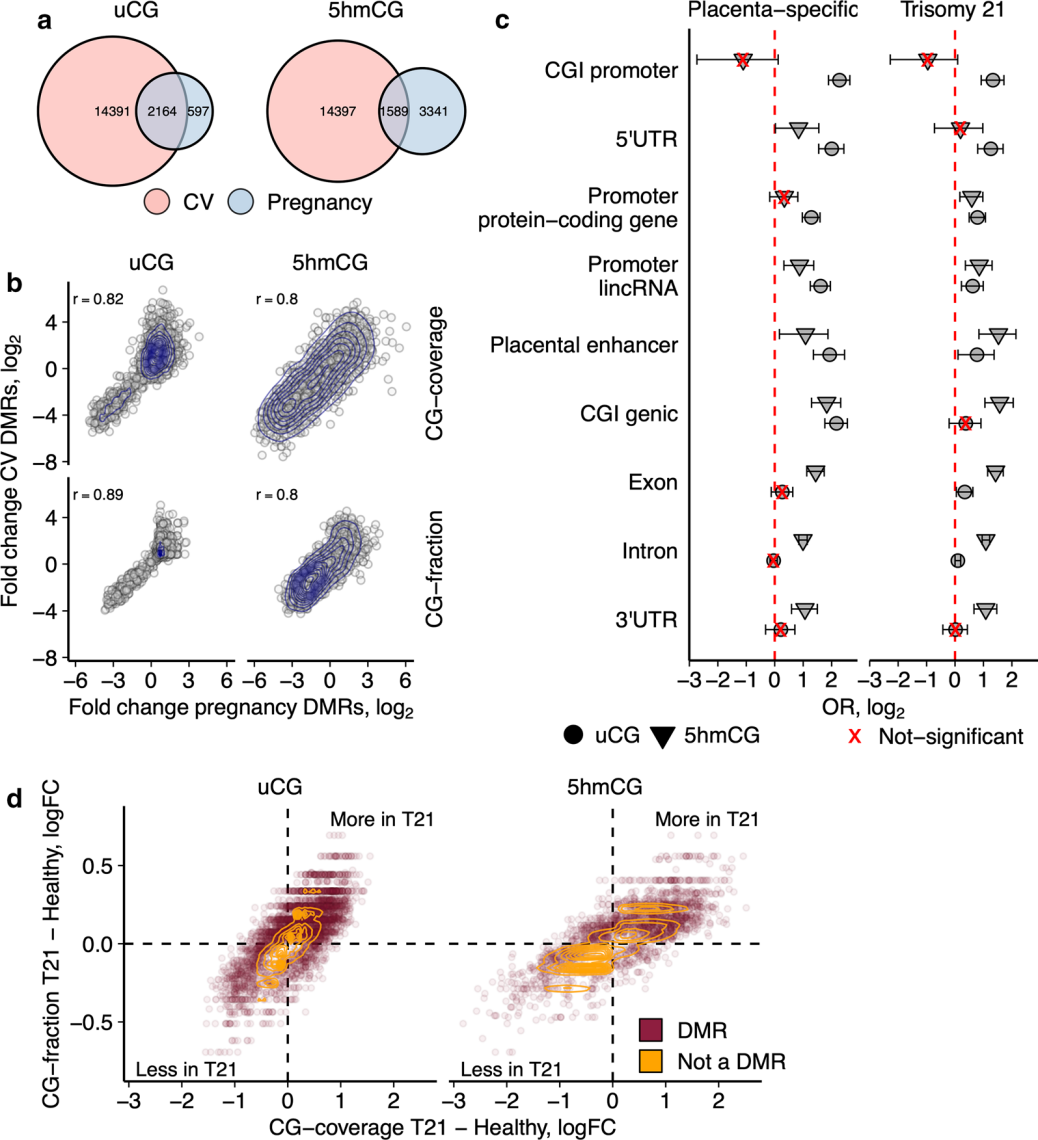
Analysis of fetal-specific and T21-specific differentially modified regions. **a** Venn diagrams indicating the overlap of the pregnancy-specific and CV-specific DMRs that constitute the placenta-specific DMR sets for uCG and 5hmCG data. **b** Pearson correlation of the modification differences observed in the placenta-specific DMRs. Pregnancy-specific changes on the x-axis indicate modification differences between NPCs and cfDNA samples of pregnant women. CV-specific changes on the y-axis indicate modification differences between NPCs and CVS. **c** Enrichment of genomic elements for the placenta-specific and T21-specific DMRs using Fisher’s exact test. CGI, CG island; lincRNA, long intergenic non-coding RNA. **d** T21-specific DMRs exhibit higher CG-coverage and CG-fraction differences than non-differentially modified regions. logFC represents a log fold-change difference between T21-diagnosed pregnancies and healthy pregnancies

First, we obtained the pregnancy-specific uCG DMRs by comparing NPCs with cfDNA samples of healthy pregnancies. Using logistic regression with the normalized CG-coverage and CG-fraction as independent variables we identified 2,761 pregnancy-specific DMRs (FDR q < 0.05). Next, by comparing NPCs and CVS we obtained 16,555 CV-specific DMRs (FDR q < 0.05; logistic regression). The same analytic approach did not yield FDR-significant DMRs from hmTOP-seq data. Therefore, we used nominal *p* < 0.05 threshold and identified 4,930 pregnancy-specific 5hmCG DMRs and 15,986 CV-specific 5hmCG DMRs. For both uCG and 5hmCG DMR sets, the overlap between the pregnancy-specific and CV-specific DMRs was larger than could be expected by chance alone. We termed the overlapping sets the placenta-specific DMRs (n = 2,164; OR = 43, and n = 1,589; OR = 5.5, for uCG and 5hmCG, respectively; *p* < 1 × 10^–16^; Fig. 4a). For the placenta-specific uCG DMRs, the difference between NPCs and cfDNA samples of pregnant women was concordant with the difference between NPCs and CV samples (r = 0.82 and r = 0.89, for CG-coverage and CG-fraction, respectively; Pearson correlation). Similar results were observed for 5hmCG DMRs (r = 0.8 and r = 0.8, for CG-coverage and CG-fraction, respectively; Pearson correlation; Fig. 4b).

To test whether the identified DMRs can be influenced by genetic variability, we calculated their overlap with methylation quantitative trait loci (mQTL) probes from ARIES database [30]. ARIES mQTL probes identified across chromosome 21 in cord blood and blood of pregnant women (birth and pregnancy mQTLs, respectively) were not significantly enriched within the DMR groups (all *p *values from Fisher’s exact test > 0.05). Moreover, both groups of mQTLs overlapped less than 1% of DMRs, irrespective of the DMR group.

The placenta-specific uCG and 5hmCG DMRs overlapped different genomic elements as could be predicted from the distinct genomic distribution of hypomethylated and 5-hydroxymethylated CGs in various tissues [21, 31]. uCG DMRs were enriched in placental enhancers, promoter CGIs, promoters of lincRNAs and protein-coding genes, and 5′UTRs. In contrast, 5hmCG DMRs were enriched in coding exons, 3′UTR and introns (Fig. 4c).

Next, we asked whether the placenta-specific DMRs are informative of fetal trisomy T21. Using leave-one-out cross-validation we constructed and evaluated a logistic regression model [32] for each placenta-specific DMR with the CG-coverage and CG-fractions as independent variables and fetal karyotype as the response variable. In total, we discovered 376 uCG and 496 5hmCG DMRs in chromosome 21 that classified the samples according to fetal karyotype with 100% accuracy (AUC = 1; Additional file 3: Table S2).

### Differential T21-specific modifications overlap known Down syndrome associated genes

Considering epigenetic changes in Down syndrome affected fetuses [33], we evaluated modification differences between cfDNA samples of healthy and T21-diagnosed pregnancies and computed the T21-specific DMRs. A logistic regression model was fitted to each 100 bp window with the CG-coverage and CG-fraction as independent variables and karyotype as the response variable, as above. In addition, we adjusted for possible confounding effects of fetal fraction and fetal sex which could not be accounted for in the previous analyses. We identified 3,490 uCG and 2,002 5hmCG DMRs (FDR q < 0.05; logistic regression), of which only 82 overlapped between the two datasets (OR = 2.3, *p* = 1.1 × 10^–10^) (Additional file 4: Table S3).

216 and 124 DMRs overlapped between the T21 and placenta-specific DMR sets for uCG and 5hmCG, respectively (OR = 6.1 and OR = 8.2, respectively; *p* < 2.2 × 10^–16^), demonstrating that different DMR identification strategies lead to different DMR sets in chromosome 21 which can be complementary for detecting fetal karyotype. The T21-affected pregnancies showed increased CG-coverage and CG-fraction across the uCG and 5hmCG DMR sets as compared to the signals in healthy pregnancies which points to increased DNA demethylation in the T21 fetuses (Fig. 4d).

Interestingly, both uCG and 5hmCG DMR datasets better overlapped the pregnancy-specific DMRs (OR = 6.6 and OR = 9, for uCG and 5hmCG, respectively) than the CV-specific DMRs (OR = 2.4 and OR = 2.9 for uCG and 5hmCG, respectively; for all comparisons *p* < 1 × 10^–16^). This result might be caused by the same tissue source of DNA or could suggest that fetal tissues other than the placenta-derived trophoblasts might contribute to the cfDNA mixture of maternal blood.

To test for a possible genotype influence, we compared the identified T21 DMR sets with birth or pregnancy mQTL loci. Both mQTL groups were not significantly enriched (Fisher’s exact test *p *value > 0.05) and covered only ~ 0.5% of identified DMRs.

Enrichment of genomic elements across the T21-specific DMRs was similar to that of the pregnancy-specific DMRs: uCG DMRs were significantly enriched towards the 5′ end of protein-coding genes (OR = 2.4, and OR = 2.5 for 5′UTR and promoter CGIs, respectively), while 5hmCG DMRs showed enrichment for genic CGIs (OR = 3) and placental enhancers (OR = 2.9) (Fig. 4c). Furthermore, distribution of the T21-specific DMRs along chromosome 21 was different for the uCG and 5hmCG datasets (*p* = 1.6 × 10^–12^; Kolmogorov–Smirnov test); most of the identified 5hmCG DMRs tended to cluster at the end of the long arm, whereas the uCG DMRs were more evenly distributed along the long arm of chromosome 21 (Additional file 2: Figure S2). 46% and 64% of the uCG and 5hmCG T21-specific DMRs, respectively, overlapped protein-coding genes. Interestingly, the above-mentioned 82 regions common for the T21-specific uCG and 5hmCG DMR sets showed very high enrichment for coding exons (OR = 4.4, *p* = 8 × 10^–4^). These exons corresponded to 7 genes, 3 of which have been previously associated with Down syndrome: *GART* [34], *DNMT3L* [35] and *AIRE* [36] (Additional file 4: Table S3). In summary, our analyses revealed widespread epigenetic changes in T21 fetuses that could be targeted for efficient prenatal diagnostics of fetal disorders from maternal cfDNA mixture.

### Detection of T21-specific differentially modified CGs by qPCR

**Fig. 5:**
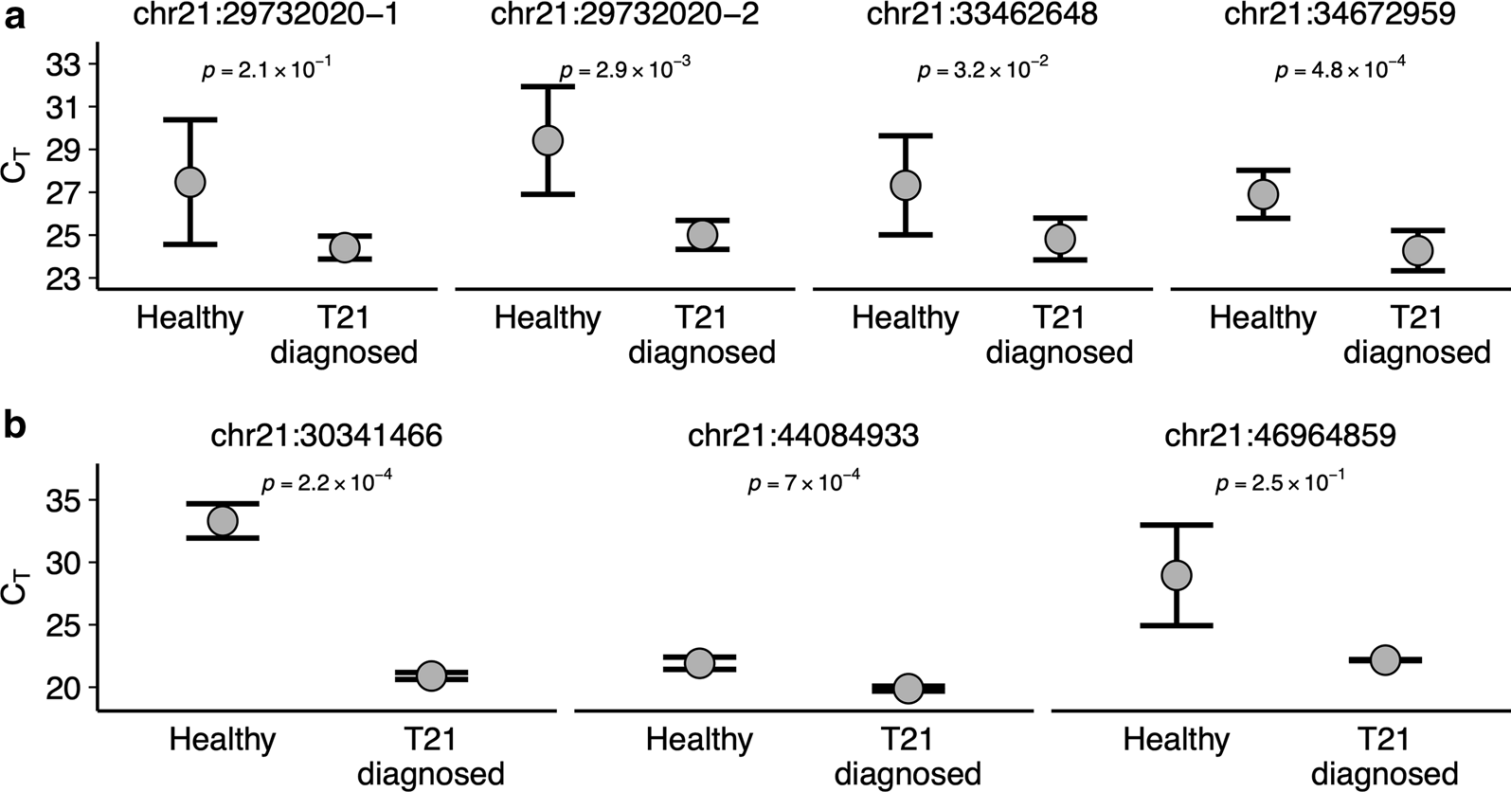
Detection of T21-specific CG-DMRs by qPCR. Relative quantification of individual T21-specific **a** uCG-DMRs and **b** 5hmCG-DMRs located on chromosome 21 using qPCR for replicated cfDNA samples of women pregnant with healthy or T21-diagnosed fetuses. The y-axis indicates the threshold cycle values (C_T_) calculated in qPCR for the regions selected from Table S4 whose genome coordinates are shown above the graphs. Numerical values of C_T_ inversely correlate to the abundance of the DMR region, indicating higher abundance of the region in the cfDNA samples of pregnant females carrying a T21-diagnosed fetus. Only samples which showed C_T_ values above the set threshold (C_T_ values ≤ 34) were included in the graphs. P-values indicated above were calculated using t-test

An investigation of differentially modified uCGs and 5hmCGs in chromosome 21 of cfDNA samples of T21-diagnosed pregnancies against the three types of control samples—CV DNA, cfDNA of healthy pregnancies and NPCs,—has led to the selection of the fetal T21-specific differentially modified individual CGs (or CG-DMRs) that could be used for detection of fetal T21. The modification state of these CG-DMRs as well as any selected CG site across the above-mentioned DMR regions can be evaluated by sequencing or, alternatively, by qPCR. We measured the modification state and enrichment of the selected T21-specific CG-DMRs (Additional file 5: Table S4) and their adjacent genomic regions in uTOP-seq and hmTOP-seq libraries by qPCR. To assess the variability in CG-DMR modification status between individuals, we applied the qPCR assay of four selected uCG-DMRs and three 5hmCG-DMRs for a group of cfDNA samples of healthy and T21-affected pregnancies which included sequenced samples and additional non-sequenced samples. Notably, all tested regions discriminated well between T21 and healthy pregnancies (Fig. 5). Overall, we demonstrated that fetal trisomy 21 can be detected by testing a single differentially modified uCG or 5hmCG site using qPCR analysis.

## Discussion

NIPT based on massive parallel sequencing of maternal cfDNA for detecting fetal chromosomal aneuploidies has already become integrated into clinical practice. Cost of testing could be significantly lowered by targeting DNA molecules of fetal origin and thus reducing the necessary analytical read count. Uncovering the presence of megabase-size placenta hypomethylated domains [13] raised an idea to employ our recently developed uTOP-seq strategy [24] for analysis of unmethylated CG sites. In this study, for the first time to our knowledge, we demonstrated the covalent derivatization and targeted sequencing of uCG sites in maternal cfDNA with the aim to detect fetal karyotype. Robust covalent labelling and high sensitivity of the uTOP-seq approach enabled detection of fetal T21 using input cfDNA amounts as low as 5 ng and only 3 million of processed sequencing reads (approx. 6 million raw reads). Such shallow sequencing depth is up to 10 times lower than that of standard whole-genome MPS-based NIPT. This is an important milestone in the future development of an affordable epigenetic NIPT test based on sequencing.

The second important milestone of our study is the first known to date demonstration that 5hmC profiling in maternal cfDNA can accurately inform on fetal karyotype. The hmTOP-seq method [25], which covalently targets 5hmC residues, enabled the construction of genome-wide 5hmC maps of relatively low 5hmC levels in CV samples and cfDNA. Most importantly, we observed that hmTOP-seq was most discriminatory in detection of T21 fetuses independently of fetal fraction. It also allowed decreasing the analytical read count down to 1 million processed reads while keeping the diagnostic accuracy at 100%. Therefore, we suggest that prenatal tests based on 5hmCG analysis could potentially maximize the diagnostic sensitivity in relation to cost and could be an optimal choice for the sequencing-based epigenetic approaches of NIPT. Furthermore, we demonstrated that fetal fraction can be measured directly from the read count of hmTOP-seq and uTOP-seq using a computational method that estimates fetal fraction independent of fetal sex.

We identified a large panel of placenta-specific uCG- and 5hmCG-biomarkers and utilized them for detection of fetal karyotype. To ascertain global methylation changes in T21 fetuses [33], we also included the computation of DMRs specific for the T21-affected fetuses. Interestingly, these DMRs better overlapped the healthy pregnancy-specific DMR sets than those of CV-specific DMRs, suggesting that DNA fragments of other tissue-origin than placenta might contribute to cffDNA. This points to a need for future comprehensive investigation of the tissue composition of maternal cfDNA. Analysis of the pregnancy-specific and T21-specific DMRs indicated the highly perturbed epigenome of T21-affected fetuses. Thus, disease-specific epigenetic characteristics should certainly be taken into account for the development of reliable NIPT of fetal aneuploidies, including T21. Importantly, we also demonstrated the informativeness of a PCR-based assay aimed at the analysis of individual CGs for testing the T21-specific modification changes.

Further validation of our approaches in a large clinical cohort is necessary. Additionally, the study needs to be expanded to other common fetal aneuploidies such as Patau and Edwards syndromes. For wider future applicability of our methods, we calculated DMRs in chromosomes 13 and 18 using the same strategy for DMR identification resulting in tissue-specific and pregnancy-specific DMR data sets (Additional file 2: Figure S3). Although the selected best DMRs demonstrated the obvious difference in signal between the sample groups, these DMRs should be validated in a clinical setting.

NIPT as a clinical application should be provided in a cost-effective and timely manner. In principle, any NIPT analysis includes three stages: sample preparation, sequencing (or qPCR) and data analysis. As library preparation time for both MPS- and TOP-seq-based protocols is comparable (up to 6 h), the number of required sequencing reads highlights the possible cost-effectiveness of the TOP-seq approaches. In contrast to whole-genome shallow coverage NIPT that requires approximately 10–20 million reads [37, 38] for high-quality karyotyping, TOP-seq allows accurate prediction of fetal karyotype with as low as 1 million single-end reads. Another advantage of TOP-seq is the simplicity of data analysis. Only a fraction (0.6–0.9 M) of total 28 million CG sites in human genome are analyzed in shallow sequencing TOP-seq. Moreover, chromosome 21 Z-score calculations were based on 10–13 thousand CG sites. Due to the relatively small number of the necessary sequencing reads per patient, the analytical pipeline does not require considerable computational resources and Z-score calculations could be done on a conventional desktop personal computer. Additional possible cost and time savings can be realized if a TOP-seq based qPCR assay is applied for NIPT. It would open the way for wider accommodation of NIPT at any lab or clinics.

## Conclusions

Although our study is limited by its sample size (48 samples in total), it demonstrated a potential of the uCG- and 5hmCG-based epigenetic analysis for NIPT of fetal aneuploidy. We detected fetal trisomy of chromosome 21 with an excellent specificity/sensitivity using both chromosome-wide data and regional modification differences. The same analytic approach may be exploited for identification of regions featuring other chromosomal, genetic or epigenetic abnormalities. Although MPS for NIPT is of higher cost than qPCR-based interrogation of selected cfDNA regions [39–41], we suggest that an affordable test based on targeted sequencing of epigenetically distinct fetal DNA could provide more information on various genetic and epigenetic abnormalities and thus, is a preferred strategy for NIPT.

## Methods

### Sample acquisition, sequencing and SeqFF

A total of 21 blood plasma samples for cfDNA from pregnant women were collected at Tartu University Hospital (Tartu, Estonia). Seven of these women were carrying fetuses with trisomy 21. All samples were obtained from the pregnant women with the gestational age of 12–20 weeks with singleton pregnancy and undergoing the low-coverage NIPT whole-genome MPS sequencing using Illumina technology as previously described [37, 42]. SeqFF was used for calculating the cell-free fetal DNA fraction for all the samples [27]. Fetal trisomy 21 was confirmed using either amniocentesis or chorionic villus sampling. In addition, blood plasma samples for cfDNA were obtained from ten non-pregnant women. Seven chorionic villi tissue samples were obtained from first trimester voluntary termination of pregnancy. cfDNA from maternal blood samples was extracted using QIAamp DNA blood Midi Kit (QIAGEN), and genomic DNA from chorionic villi tissue and blood was extracted by phenol–chloroform extraction.

### cfDNA and CV DNA processing by uTOP-seq and hmTOP-seq

In uTOP-seq, 4–10 ng of cfDNA (or 100 ng of CV tissue DNA, sheared to 200 bp by Covaris sonicator) were labeled with 0.11 μM eM.SssI [23] in 10 mM Tris–HCl (pH 7.4), 50 mM NaCl, 0.5 mM EDTA buffer supplemented with 200 μM Ado-6-azide cofactor [43] for 1 h at 30 °C followed by thermal inactivation at 65 °C for 20 min and Proteinase K treatment (0.2 mg/ml) for 30 min at 55 °C and finally column purified (GeneJET PCR purification kit, Thermo Fisher Scientific (TS)). In hmTOP-seq, 5hmC glycosylation was carried out with 5–10 ng of cfDNA (or 200 ng of CV tissue DNA, sheared to 200 bp by Covaris sonicator) supplemented with 50 µM UDP-6-azide-glucose (Jena Bioscience) and 2.5–5 U T4 β-glucosyltransferase (TS) for 1 h 37 °C followed by enzyme inactivation at 65 °C for 20 min and column purification (GeneJET PCR Purification kit (TS)). After ligation of the partially complementary adapters as described previously (step 2, [24]), covalently labeled DNA was supplemented with 20 µM alkyne-containing DNA oligonucleotide (which was biotinylated for 5hmC analysis) (5′-T(alkyneT)TTTTGTGTGGTTTGGAGACTGACTACCAGATGTAACA-(biotin)-3′, Base-click) and 8 mM CuBr: 24 mM THPTA mixture (Sigma) in 50% of DMSO, incubated for 20 min at 45 °C and subsequently diluted to < 1.5% DMSO before column purification (GeneJET NGS Cleanup Kit, Protocol A (TS)). DNA recovered after the biotinylation step was incubated with 0.1 mg Dynabeads MyOne C1 Streptavidin (TS) in buffer A (10 mM Tris–HCl (pH 8.5), 1 M NaCl) at room temperature for 3 h on a roller. DNA-bound beads were washed 2 × with buffer B (10 mM Tris–HCl (pH 8.5), 3 M NaCl, 0.05% Tween 20); 2 × with buffer A (supplemented with 0.05% Tween 20); 1 × with 100 mM NaCl and finally resuspended in water and heated for 5 min at 95 °C to recover the enriched DNA fraction. Purified DNA after oligonucleotide conjugation (uCG) or biotin-enrichment (5hmC) was subsequently used in a priming reaction with 1 U Pfu DNA polymerase (TS), 0.2 mM dNTP, 0.5 μM complementary priming strand (EP; 5′-TGTTACATCTGGTAGTCAGTCTCCAAACCACACAA-3′, with custom LNA modifications (Exiqon) and phosphorothioate linkages at the 3′ end). Priming reaction mixture was incubated at the following cycling conditions: 95 °C 2 min; 5 cycles at 95 °C 1 min, 65 °C 10 min, 72 °C 10 min. Amplification of a primed DNA library was carried out by adding the above reaction mixture to 100 μl of amplification reaction containing 50 µl of 2 × Platinum SuperFi PCR Master Mix (TS) and barcoded fusion PCR primers A(Ad)-EP-barcode-primer (63 nt) and trP1(Ad)-A2-primer (45 nt) at 0.5 μM each (both primers contained phosphorothioate modifications). Thermocycler conditions: 94 °C 4 min; 15 cycles (uCG cfDNA), or 17 cycles (5hmC cfDNA), or 12 cycles for CV DNA at 95 °C 1 min, 60 °C 1 min, 72 °C 1 min. The final libraries were size-selected for ~ 270 bp fragments (MagJET NGS Cleanup and Size Selection Kit, (TS)), and their quality and quantity were tested on 2100 Bioanalyzer (Agilent). Libraries were subjected to Ion Proton (TS) sequencing.

### Processing of sequencing data

Raw uTOP-seq and hmTOP-seq sequencing reads were processed as described in [24] except for the 3′ sequence ends, where adapter sequences were trimmed only if they were identified using cutadapt with maximum allowed error rate 0.1 [44]. Processed reads were mapped to reference human genome version hg19 and coverage for each CG dinucleotide was computed as the total number of reads starting from or within 3 bp of the dinucleotide on either of the strands. On average, 40% of raw reads were retained for downstream analysis per sample.

Outlier identification was performed separately for uCG and 5hmC samples. CG coverage matrices were transformed using Hellinger transformation [45] and then represented in two-dimensional space using non-metric multidimensional scaling (nMDS) with Bray–Curtis similarity index [46]. Samples that were further than two standard deviations away from the mean of their own sample group (cfDNA of NPCs, pregnancy cfDNA, CVS) in either nMDS1 or nMDS2 dimension were deemed outliers and removed from further analysis. There were three outlying samples in uCG and one in 5hmCG dataset: two in 20 uCG cfDNA samples, one in 7 uCG CV samples and one 5hmCG sample was removed from 21 5hmCG cfDNA samples (including the samples of shallow sequencing).

### Karyotype detection from uCG and 5hmCG data

Chromosome-wide coverage was defined as the sum of uTOP-seq or hmTOP-seq coverage across all identified CGs pertaining to a chromosome. A CG-fraction was defined as the ratio of CG dinucleotides covered by at least one uTOP-seq or hmTOP-seq read to the total number of CGs in a chromosome. Z-scores were calculated following [29]. Briefly, for a sample *i,* value *X*_*i,21/r*_ was defined as the ratio of signal (either CG-coverage or identified CG-fraction) between chromosome 21 and a reference chromosome, *r.* We then used *X*_*i,21/r*_ to compute the mean, *μ*, and standard deviation, *σ,* of the healthy control subpopulation of samples. Then, for each sample *i*, we calculated Z-score as *Z*_*i, 21/r*_ = *(X*_*i.21/r *_− *μ)/σ*. We found that the results were most stable and consistent, when chromosome 20 and chromosome 16 for uCG and 5hmCG data, respectively, were used as references.

In silico subsampling of libraries was performed by randomly selecting a set of processed reads that have been assigned to a CG site. For each subset size, 30 subsampled datasets were produced and karyotype prediction models were trained and evaluated on each of them. Model training and evaluation was performed using leave-one-out cross-validation. For each cross-validation cycle one sample was set aside and a logistic regression model was built using the Z-scores computed on the remaining samples. In 5hmCG data, when subset size was equal or below one million reads, we also included the shallow sequencing samples (2.5 M raw reads) into the evaluation loop. The model was then used to predict the karyotype of the retained sample. Once the karyotypes of all samples were obtained, the accuracy of prediction was evaluated as the area under the receiver-operator curve (AUC). Area AUC = 1 (i.e. 100%) indicates perfect classification accuracy.

### Identification of differentially modified regions

The chromosome 21 was partitioned into 100-bp non-overlapping windows. For each window log transformed CG-coverage and CG-fraction was calculated. CG-coverage was normalized by the total read count in a reference chromosome. CG-fraction was normalized by the overall identified fraction in a reference chromosome. Chromosomes 20 and 16 were used as references for uCG and 5hmCG data, respectively. Next, for each window two logistic regression models were fitted. Full model included CG-coverage, CG-fraction, and, for T21-specific DMRs, fetal sex and fetal fraction, as independent variables. CG-coverage and CG-fraction were excluded from the null model. ANOVA Chi-squared test was used to compare full and null models to obtain *p* value. In cases where models did not converge, fetal sex was removed. FDR was used to adjust *p* values for multiple testing and q < 0.05 was used as a significance threshold, if not specified otherwise.

For each placenta-specific DMR, leave-one-out cross-validation procedure was performed as described above in order to determine its ability to diagnose T21. For each cross-validation cycle, Bayesian generalized linear model [32] with normalized CG-coverage and CG-fraction as independent variables was constructed. DMRs with AUC = 1 were selected as discriminatory of fetal karyotype.

### Enrichment analyses

Enrichment of genomic elements with the strongest signal was calculated as follows. First, the genome was divided into 1 kb-wide non-overlapping windows. Within each window total coverage was computed per sample. The total coverage values were then averaged per group of samples (cfDNA of NPCs, pregnancy cfDNA samples, CVS) and windows falling among top 10% most covered windows were designated as those having the highest signal. Then, contingency table was computed for each CG falling into one of the highest signal windows and overlapping a genomic region. Fisher’s exact test was performed to estimate the odds ratio and *p* value.

Enrichment of DMRs with genomic regions was computed by forming a contingency table which contained information whether each DMR is significant and overlaps genomic regions of interest. As above, Fisher’s exact test was used to estimate the odds ratios and *p *values. Placental enhancer regions were downloaded from the enhancer atlas [47]. Gene annotations were downloaded from the GENCODE genes and promoters were defined as regions 2 kb upstream from the gene start [48]. Human genome annotation (build hg19), CGI and repeat datasets were downloaded from the UCSC database (https://genome.ucsc.edu).

Birth and pregnancy mQTLs were retrieved from the ARIES mQTL database followed by the selection of only high-quality probes [30, 49]. In total, there were 4243 Illumina Infinium HM450 array probes in chromosome 21 and 2642 after selecting only high-quality probes (238 birth mQTL and 291 pregnancy mQTLs). Enrichment of mQTL probes with DMRs was calculated by creating a contingency table which evaluated whether each Illumina Infinium HM450 array probe is an mQTL and overlaps a DMR.

Chromosome 21 ideogram was plotted using karyoploteR R package [50]. Computational analyses were performed using R version 3.5 [51].

### Quantitative liquid chromatography coupled with tandem mass spectrometry (HPLC–MS/MS) analysis of genomic DNA

100–500 ng of blood and CV tissue genomic DNA [n = 2 CV; n = 3 blood (with two technical replicates)] samples were digested with 0.5 U Nuclease P1 (Sigma) for 2 h at 55 °C in 40 µl of P1 buffer, then dephosphorylated by adding 1 μl FastAP (TS) phosphatase and incubated overnight at 37 °C. Samples were analyzed on an integrated HPLC/ESI-QQQ system (Agilent) equipped with a Supelco Discovery®HS C18 column (7.5 cm × 2.1 mm, 3 μm) by elution with a linear gradient of solvents A (0.0075% formic acid in water) and B (0.0075% formic acid in acetonitrile) at a flow of 0.3 ml/min at 30 °C as follows: 0–6 min, 0% B; 6–18 min, 10% B; 18–20 min, 100% B. Mass spectrometer was operating in the positive ion MRM mode and intensities of nucleoside-specific ion transitions were recorded: d5mC m/z 242.1 → 126.1; d5hmC m/z 258.1 → 142.1; dG m/z 268.1 → 152.1. Ionization capillary voltage was set to 1800 V, drying gas temperature 300 °C and flow rate 10 l/min, collision energy 15 V. Standard d5mC, d5hmC and dG nucleosides (Trilink Biotech) were used for external calibration. d5hmC calibration curves were constructed by plotting ion counts against different concentrations of d5hmC standards and linear plots were obtained with *R*^2^ values of 0.999. To account for input DNA differences, DNA sample normalization according to dG was performed. Data was analysed using Agilent MassHunter software and Microsoft Excel.

### Detection of fetal trisomy T21 by qPCR

The difference in labeling intensity at specific CG-DMRs, shown in Additional file 5: Table S4, was tested in female cfDNA carrying healthy or T21-diagnosed fetuses. 0.5 ng of the final uTOP-seq and hmTOP-seq libraries were used for measurement of the labeling intensity of uCGs and 5hmCGs by qPCR with a Rotor-Gene Q real-time PCR system (Qiagen) using Maxima Sybr Green/ROX qPCR Master Mix (TS). 0.3 mM of each primer pair (shown in Additional file 5: Table S4) was used in each reaction, wherein one of the primers binds to a genomic region nearby a CG site, and another primer binds in vicinity of the same CG to allow PCR amplification of the region to occur. The amplification conditions were set as: 95 °C for 10 min, 40 cycles 95 °C for 15 s, 60 °C for 60 s. Due to high methylation and absence of hydroxymethylation of the selected CG-DMRs in healthy pregnancy cfDNA samples, these samples often do not show the respective amplification product (no or ambiguous products on electrophoresis gels and in melting curves). Therefore, the loci which amplify above the experimental values of C_T_ 34 (C_T_ values ≤ 34) were treated as detected in a qPCR test and only samples which showed C_T_ values above the set threshold were included in Fig. 5.

## Supplementary information

**Additional file 1: Table S1.** DNA sample information.

**Additional file 2: Figure S1.** Detection of fetal trisomy T21 using CG-coverage. **(a)** Effect of fetal fraction on the Z-score indicating fetal karyotype. Total normalized chromosome 21 coverage was used to compute Z-scores. Dashed lines represent logistic regression fits. 100% diagnostic accuracy of fetal trisomy T21 computed using CG-coverage of chromosome 21 is independent of fetal fraction. hmTOP-seq samples of shallow sequencing (2.5 million reads) are indicated with red circles. **(b)** Effect of a reduced library size on classification accuracy. 100% diagnostic accuracy is achieved using 5 million or 1 million of processed sequencing reads in uTOP-seq and hmTOP-seq, respectively. Reads were randomly sampled and karyotype determined using a leave-one-out cross-validation. In each cross-validation loop a logistic regression model was built with Z-scores computed from normalized chromosome coverage. Error bars indicate the standard deviation from mean AUC across 30 sampling iterations. **Figure S2.** Distribution of T21-specific DMR. Ideogram of chromosome 21 (centromeric region marked in red) showing distributions of the T21-specific uCG and 5hmCG DMRs. DMRs shared between the sets are indicated with dark vertical bars. 7 genes containing the shared DMRs across their exons are specified above the graph. **Figure S3.** Difference in (a) uCG and (b) 5hmCG signal across the selected DMRs identified for chromosome 13 and chromosome 18 between CV tissue DNA of the 1st trimester fetuses and cfDNA samples of NPCs; and between NPCs and pregnant female carrying a healthy fetus. The genomic coordinates of DMRs are shown above the graphs. For chromosome 13, we obtained 1394 pregnancy-specific uCG DMRs (FDR q < 0.05) and 25,091 tissue-specific uCG DMRs (FDR q < 0.05; logistic regression) and using nominal *p* < 0.05 threshold, 4255 pregnancy-specific 5hmCG DMRs and 22,526 tissue-specific 5hmCG DMRs. For chromosome 18, we obtained 1321 pregnancy-specific uCG DMRs (FDR q < 0.05), 22,121 tissue-specific uCG DMRs (FDR q < 0.05; logistic regression) and 3626 pregnancy-specific 5hmCG DMRs and 20,780 tissue-specific 5hmCG DMRs.

**Additional file 3: Table S2.** List of the identified DMRs with AUC = 1.

**Additional file 4: Table S3.** T21-specific DMRs common for uCG and 5hmCG data.

**Additional file 5: Table S4.** Selected fetal T21-specific CG-DMRs.

## Data Availability

Raw and processed uTOP-seq and hmTOP-seq data of CV and cfDNA samples have been deposited in the NCBI Gene Expression Omnibus under accession number GSE148964. Some datasets supporting the conclusions of this article are included within the article and its additional files. Other data supporting the results reported in the article is available from the corresponding author upon a reasonable request.

## Abbreviations

AUC: Area under the curve
cfDNA: Cell-free DNA
cffDNA: Cell-free fetal DNA
CGI: CG island
C_T_: Threshold cycle value
CV: Chorionic villi
CVS: Chorionic villi tissue sample
DMR: Differentially modified region
FC: Fold change
FDR: False discovery rate
5hmC: 5-Hydroxymethylcytosine
5hmCG: 5-Hydroxymethylated CG dinucleotide
hmTOP-seq: 5hmC-specific tethered oligonucleotide–primed sequencing
HPLC–MS/MS: Quantitative liquid chromatography coupled with tandem mass spectrometry
M: Million
MeDIP: Methyl-DNA immunoprecipitation
MPS: Massive parallel sequencing
mQTL: Methylation quantitative trait loci
NIPT: Non-invasive prenatal testing
nMDS: Non-metric multidimensional scaling
NPC: Non-pregnant control
OR: Odds ratio
T21: Trisomy of chromosome 21
TOP-seq: Tethered oligonucleotide-primed sequencing
uCG: Unmodified CG site
uTOP-seq: uCG-specific tethered oligonucleotide–primed sequencing
