# Supplementary material for: Identification of fetal unmodified and 5-hydroxymethylated CG sites in maternal cell-free DNA for non-invasive prenatal testing
Source: Clin Epigenetics. 2020 Oct 20;12:153. doi: 10.1186/s13148-020-00938-x (PMC7574562; doi:10.1186/s13148-020-00938-x)
Supplement: Supplementary file 2 — Additional file 2: Figure S1. Detection of fetal trisomy T21 using CG-coverage. (a) Effect of fetal fraction on the Z-score indicating fetal karyotype. Total normalized chromosome 21 coverage was used to compute Z-scores. Dashed lines represent logistic regression fits. 100% diagnostic accuracy of fetal trisomy T21 computed using CG-coverage of chromosome 21 is independent of fetal fraction. hmTOP-seq samples of shallow sequencing (2.5 million reads) are indicated with red circles. (b) Effect of a reduced library size on classification accuracy. 100% diagnostic accuracy is achieved using 5 million or 1 million of processed sequencing reads in uTOP-seq and hmTOP-seq, respectively. Reads were randomly sampled and karyotype determined using a leave-one-out cross-validation. In each cross-validation loop a logistic regression model was built with Z-scores computed from normalized chromosome coverage. Error bars indicate the standard deviation from mean AUC across 30 sampling iterations. Figure S2. Distribution of T21-specific DMR. Ideogram of chromosome 21 (centromeric region marked in red) showing distributions of the T21-specific uCG and 5hmCG DMRs. DMRs shared between the sets are indicated with dark vertical bars. 7 genes containing the shared DMRs across their exons are specified above the graph. Figure S3. Difference in (a) uCG and (b) 5hmCG signal across the selected DMRs identified for chromosome 13 and chromosome 18 between CV tissue DNA of the 1st trimester fetuses and cfDNA samples of NPCs; and between NPCs and pregnant female carrying a healthy fetus. The genomic coordinates of DMRs are shown above the graphs. For chromosome 13, we obtained 1394 pregnancy-specific uCG DMRs (FDR q < 0.05) and 25,091 tissue-specific uCG DMRs (FDR q < 0.05; logistic regression) and using nominal p < 0.05 threshold, 4255 pregnancy-specific 5hmCG DMRs and 22,526 tissue-specific 5hmCG DMRs. For chromosome 18, we obtained 1321 pregnancy-specific uCG DMRs (FDR q < 0.05), 22,121 tiss [file 13148_2020_938_MOESM2_ESM.pdf]

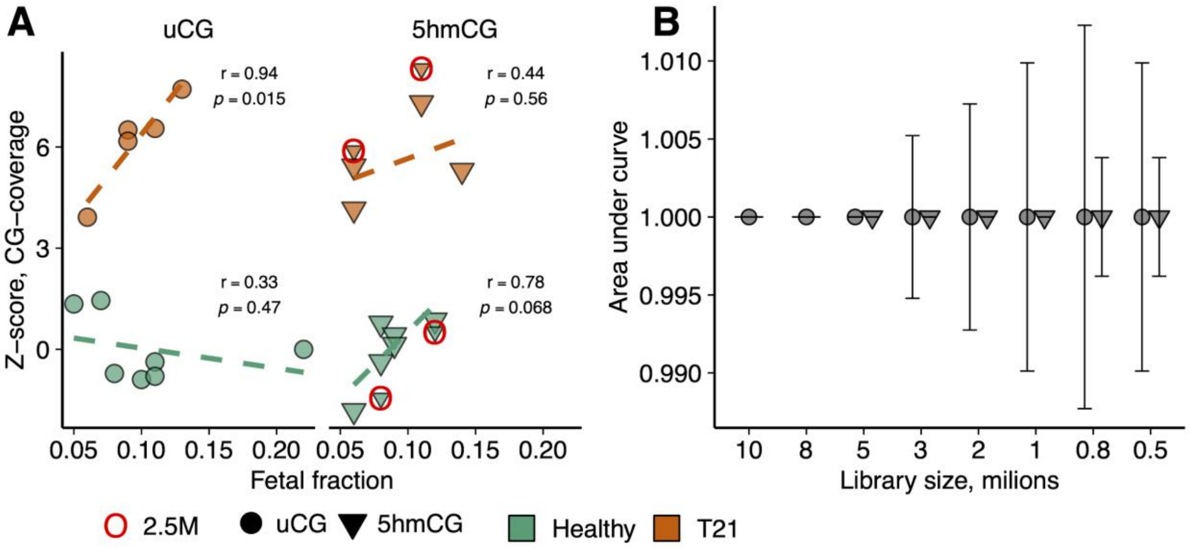

Figure S1. Detection of fetal trisomy T21 using CG-coverage. **(a)** Effect of fetal fraction on the Z-score indicating fetal karyotype. Total normalized chromosome 21 coverage was used to compute Z-scores. Dashed lines represent logistic regression fits. 100% diagnostic accuracy of fetal trisomy T21 computed using CG-coverage of chromosome 21 is independent of fetal fraction. hmTOP-seq samples of shallow sequencing (2.5 million reads) are indicated with red circles. (**b**) Effect of a reduced library size on classification accuracy. 100% diagnostic accuracy is achieved using 5 million or 1 million of processed sequencing reads in uTOP-seq and hmTOP-seq, respectively. Reads were randomly sampled and karyotype determined using a leave-one-out cross-validation. In each cross-validation loop a logistic regression model was built with Z-scores computed from normalized chromosome coverage. Error bars indicate the standard deviation from mean AUC across 30 sampling iterations.

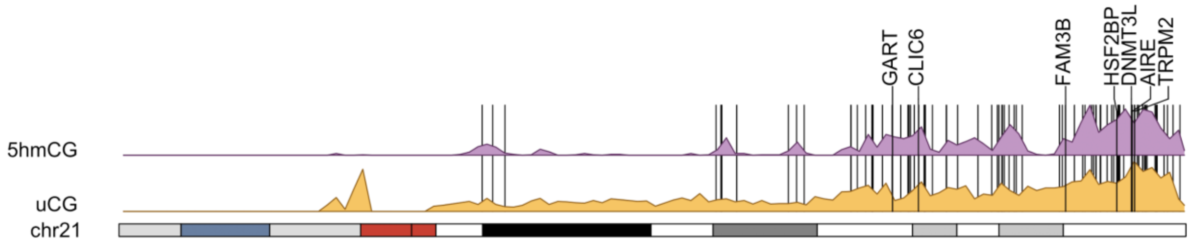

Figure S2. Ideogram of chromosome 21 (centromeric region marked in red) showing distributions of the T21-specific uCG and 5hmCG DMRs. DMRs shared between the sets are indicated with dark vertical bars. 7 genes containing the shared DMRs across their exons are specified above the graph.

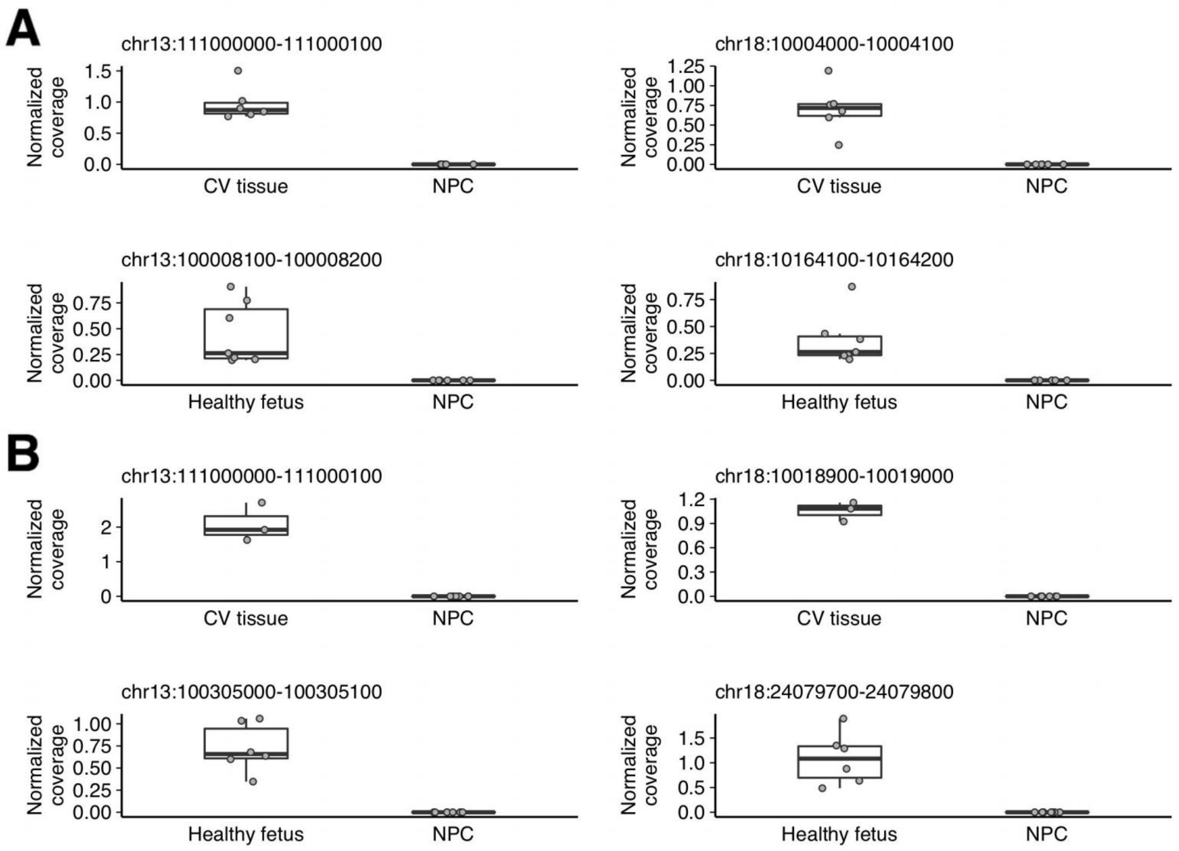

Figure S3. Difference in (a) uCG and (b) 5hmCG signal across the selected DMRs identified for chromosome 13 and chromosome 18 between CV tissue DNA of the 1st trimester fetuses and cfDNA samples of NPCs; and between NPCs and pregnant female carrying a healthy fetus. The genomic coordinates of DMRs are shown above the graphs. For chromosome 13, we obtained 1,394 pregnancy-specific uCG DMRs (FDR $q < 0.05$) and 25,091 tissue-specific uCG DMRs (FDR $q<0.05$; logistic regression) and using nominal $p<0.05$ threshold 4,255 pregnancy-specific 5hmCG DMRs and 22,526 tissue-specific 5hmCG DMRs. For chromosome 18, we obtained 1,321 pregnancy-specific uCG DMRs (FDR $q<0.05$), 22,121 tissue-specific uCG DMRs (FDR $q<0.05$; logistic regression) and 3,626 pregnancy-specific 5hmCG DMRs and 20,780 tissue-specific 5hmCG DMRs.
